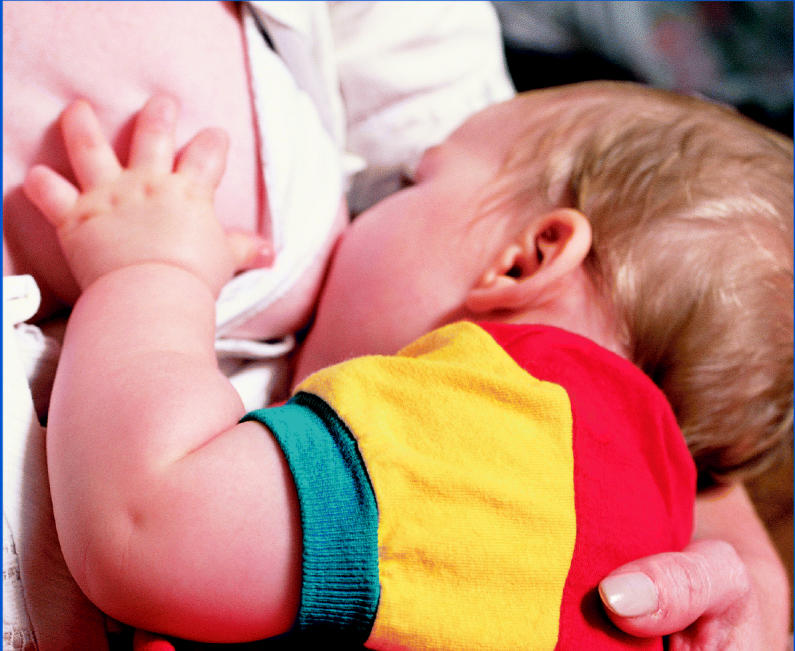# Headliners: Childhood Caner: Breastfeeding and the Risk of Childhood Leukemia

**Published:** 2005-02

Kwan ML, Buffler PA, Abrams B, Kiley VA. 2004. Breastfeeding and the risk of childhood leukemia: a meta-analysis. Public Health Rep 119:521–535.

Leukemia is the leading cause of cancer deaths in children under age 15 in the United States. Acute lymphoblastic leukemia (ALL) accounted for 78% of all U.S. childhood leukemia cases diagnosed from 1975 to 1995, while acute myeloid leukemia (AML) accounted for 16%, according to data from the Surveillance, Epidemiology, and End Results registry. Recent studies have suggested that breastfeeding may protect children from developing ALL. NIEHS grantee Patricia Buffler and colleagues at the University of California, Berkeley, School of Public Health performed a meta-analysis of the scientific literature to investigate the effect of short-term and long-term breastfeeding on the risk of childhood ALL and AML. Their results suggest a statistically significant protective effect for both short- and long-term breastfeeding.

The California team used a fixed effects model to systematically combine the results of 14 case–control studies addressing the effect of short-term (6 months or less) and long-term (longer than 6 months) breastfeeding on the risk of childhood ALL and/or AML. The 14 articles contributed 6,835 cases of ALL and 1,216 cases of AML. The researchers also analyzed studies that both did and did not adjust for socioeconomic status of the child’s family, a possible risk factor for childhood leukemia.

The results showed that children who breastfed longer than 6 months had a 24% reduced risk of ALL and a 15% reduced risk of AML. Short-term breastfeeding, similarly, was protective for ALL (12% reduced risk), although the observed 10% reduction in risk for AML was not statistically significant. Results for the two subgroups of studies that considered/did not consider socioeconomic status were not significantly different from the results for the 14 studies combined. Thus, socioeconomic status had minimal influence on the breastfeeding results, say the authors.

The authors note that “the potential protective effect of breastfeeding . . . may be more complicated than the current literature suggests.” Nevertheless, they conclude, the available evidence indicates that, despite caveats, such a protective effect likely does exist.

## Figures and Tables

**Figure:**